# A random survey of the prevalence of falsified and substandard antibiotics in the Lao PDR

**DOI:** 10.1093/jac/dkab435

**Published:** 2022-05-29

**Authors:** Patricia Tabernero, Isabel Swamidoss, Mayfong Mayxay, Maniphone Khanthavong, Chindaphone Phonlavong, Chanthala Vilayhong, Chanvilay Sichanh, Sivong Sengaloundeth, Michael D. Green, Paul N. Newton

**Affiliations:** 1 Lao-Oxford-Mahosot Hospital-Wellcome Trust Research Unit (LOMWRU), Microbiology Laboratory, Mahosot Hospital, Vientiane, Lao PDR; 2 Public Health Unit, Faculty of Medicine, University of Alcalá, Alcalá de Henares, Spain; 3 Division of Parasitic Diseases and Malaria, U.S. Centers for Disease Control and Prevention (CDC), Atlanta, Georgia, USA; 4 Centre for Tropical Medicine & Global Health, Nuf?eld Department of Medicine, Churchill Hospital, University of Oxford, Oxford, UK; 5 Institute of Research and Education Development, University of Health Sciences, Vientiane, Lao PDR; 6 Centre of Malariology, Parasitology and Entomology (CMPE), Vientiane, Lao PDR; 7 Bureau of Food and Drug Inspection (BFDI), Ministry of Health, Government of the Lao PDR, Vientiane, Lao PDR; 8 Food and Drug Department (FDD), Ministry of Health, Government of the Lao PDR, Vientiane, Lao PDR; 9 Infectious Diseases Data Observatory, Nuf?eld Department of Medicine, Churchill Hospital, University of Oxford, Oxford, UK; 10 Faculty of Infectious and Tropical Diseases, London School of Hygiene and Tropical Medicine (LSHTM), London, UK

## Abstract

**Objectives:**

In 2012, a stratified random survey, using mystery shoppers, was conducted to investigate the availability and quality of antibiotics sold to patients in the private sector in five southern provinces of the Lao People’s Democratic Republic (Laos).

**Methods:**

A total of 147 outlets were sampled in 10 districts. The active pharmaceutical ingredient (API) content measurements for 909 samples, including nine APIs (amoxicillin, ampicillin, ceftriaxone, ciprofloxacin, doxycycline, ofloxacin, sulfamethoxazole, tetracycline and trimethoprim), were determined using HPLC.

**Results:**

All the analysed samples contained the stated API and we found no evidence for falsification. All except one sample had all the units tested with %API values between 75% and 125% of the content stated on the label. However, we identified the presence of substandard antibiotics: 19.6% (201/1025) of samples had their units outside the 90%–110% content of the label claim and 18.3% (188/1025) of the samples had units outside the International Pharmacopoeia/United States Pharmacopoeia assay (percentage of label claim) specifications. Trimethoprim had a high number of samples [51.6% (64)] with units below the limit range, followed by ceftriaxone [42.9% (3)] and sulfamethoxazole [34.7% (43)]. Doxycycline, ofloxacin and ciprofloxacin had the highest number of samples with high API content: 43.7% (38), 14.7% (10) and 11.8% (2), respectively. Significant differences in %API were found between stated countries of manufacture and stated manufacturers.

**Conclusions:**

With the global threat of antimicrobial resistance on patient outcomes, greater understanding of the role of poor-quality antibiotics is needed. Substandard antibiotics will have reduced therapeutic efficacy, impacting public health and control of bacterial infections.

## Introduction

Access to good-quality medicines is a critical factor for the effective management and control of diseases globally and universal health coverage.^1^ The increased accessibility and inappropriate use of antimicrobials have led to enhanced selective pressure and development of resistant pathogens. Antimicrobial resistance (AMR) threatens the effective prevention and treatment of infections in both the developed and developing world, and is growing at an alarming pace.^2–5^

Factors contributing to the development of antibiotic resistance include inappropriate use of antibiotics due to poor prescribing and patient adherence. A neglected factor has been poor-quality medicines, including those that are falsified or are substandard due either to errors in manufacture or degradation while in the supply chain. The latter two categories are included together in the 2017 WHO definition of substandard.^6^ Poor storage conditions may result in physicochemical changes causing degradation or altered dissolution of active ingredients. Subtherapeutic amounts of active pharmaceutical ingredients (APIs) and poor API dissolution of antimicrobials engender resistance for some pathogens.^7^ Resistance is most likely to develop when pathogens are exposed to low API concentrations, high enough to kill susceptible organisms, but not resistant ones.^7–10^

Although there is a logical relationship, quantification of the contribution made by poor-quality medicines to AMR remains unknown due to the lack of information and understanding. Few objective data on the prevalence of poor-quality antibiotics exist. Some research groups and international non-governmental organizations (NGOs) have tried, over the last two decades, to estimate their prevalence and highlight the problem in developing countries.^11–21^ Examples include a study conducted in the late 1990s in Nigeria and Thailand, showing that 36% of the samples collected in Nigeria and 40% of samples collected in Thailand contained quantities of APIs outside pharmacopoeial limits.^22^ The death of a patient from bacterial meningitis was associated with substandard ceftriaxone in Uganda in 2013.^14^ The strong demand for antibiotics runs the risk of creating a market for falsification,^13^ and they are widely falsified.^11^ There is increasing awareness that poor-quality medicines are important impediments to public health.^15,23–26^

The problem is aggravated by the lack of testing facilities in low- and middle-income countries and poor data sharing.^27^ Misdiagnosis and AMR are usually considered the main reasons for treatment failure, without consideration of the risk of poor-quality medicines (and, consequently, investigations on the latter are not prompted).

Reports in the Greater Mekong subregion have demonstrated high frequencies of falsified and substandard antimalarials,^25,28–33^ but there are few data on antibiotic quality in the public domain. Surveys conducted in the late 1990s investigated the availability of poor-quality antibiotics including ampicillin and tetracycline in the Lao People’s Democratic Republic (PDR) (Laos).^31,34^ Operation Storm I and II, conducted in 2008–09, showed that 31% of seized antibiotics analysed were of poor quality.^35^

However, very few data were acquired using random sampling that would allow objective estimates of the proportion of a nation’s or a province/region’s antibiotic supply that is substandard or falsified, and there are none from Laos.^36–38^ Data on antibiotic resistance in Laos are scarce, but ESBL Enterobacteriaceae are becoming more frequent.^39–41^

In 2012, we conducted a survey to investigate the availability and quality of selected antimalarials and antibiotics sold to patients in the private sector in five southern Lao provinces. The study methodology and results for antimalarials have been described,^37^ and here we report the results on the quality of the antibiotics collected.

## Methods

### Setting

Laos has a population of ∼ 6.8 million people, with the majority (60.3%) living in rural areas.^42–45^ One urban and one rural district (i.e. stratified by urbanization) were selected using simple random selection, by random number tables, from each of the five southern provinces, and all known outlets in these districts were sampled.^37^ The districts selected were Adsaphangthong and Sepon in Savannakhet Province, Salavan and Toumlane in Salavan Province, Sekong and Thateng in Sekong Province, Sammakkyxay and Sanamxay in Attapeu Province, and Pakse and Sanasoumboun in Champasak Province (Figure S1, available as Supplementary data at *JAC* Online).

### Study design

A cross-sectional random sampling of private sector medicine outlets was conducted in the five southern Lao provinces starting in September 2012, lasting 4 weeks. One male and one female research assistant from Vientiane purchased, as mystery shoppers, the anti-infective medicines from all private retail outlets identified in the selected districts. Prior to the survey, a 1 week training was conducted in Vientiane. This included pre-testing of the data collection tools and the debriefing process.

Outlets were visited twice, first by a mystery shopper who stated to be a friend of a sick malaria patient working in construction in southern Lao (for the antimalarial medicine quality survey^37^) and, secondly, by another mystery shopper with a handwritten list of essential antibiotics and anti-TB medicines (Text S1). Visits were conducted on successive days. Twenty tablets/capsules of each preparation of amoxicillin, ampicillin, ciprofloxacin, co-trimoxazole, ofloxacin, tetracycline and doxycycline were requested. Rifampicin, isoniazid, ethambutol and pyrazinamide were also requested.

If no staff were present at the first visit, two further attempts were made to visit outlets. Hand-held GPS (Global Positioning System) units were used to map outlet locations (within ±10 m). Debriefing of the mystery shopper was conducted after each outlet interaction on the same day using a semi-structured questionnaire (Form S1).

### Inclusion criteria

All private pharmacies, private clinics or medicine sellers in the study districts, whether registered or unregistered, were eligible for inclusion in the survey. Private pharmacies are classified depending on the qualifications of the licensee:^31,46^ class I pharmacies are run by a qualified pharmacist with a university degree; class II pharmacies are run by an assistant pharmacist; and class III pharmacies are run by any medical professional, usually an auxiliary nurse or a low-level pharmacist. Registered Private Clinics are run by medical doctors outside Government working hours. These are licensed to sell antibiotics.

Poor-quality medicines were defined as falsified or substandard based on WHO definitions without consideration of intellectual property issues.^6^ A sample was defined as a group of apparently physically identical dosage units (e.g. tablets or vials), from one brand and one batch obtained at the same time from the same outlet. Samples were kept in a foam box and sent to Vientiane within 3–4 days of collection, for storage in a refrigerator at +4°C before shipment for analysis.

All data were double-entered in a pre-established Epi-Data database. Data were analysed using STATA (v11.2, Stata Corp, College Station, TX, USA), RStudio Version 1.0.136 (RStudio Team, 2016) and Microsoft Excel.

### Laboratory analysis of the samples

Anti-infective samples were sent for chemical analysis to the CDC in Atlanta, USA. Analysis was completed 24 months after sample collection. The API content measurements for each sample were determined using HPLC.^47^ Between one and three dosage units were tested for each sample (when available) and the mean of the percentage API (%API), with reference to the stated dose on the packaging, was calculated.

Samples were primarily classified as meeting the quality requirements if the amount of API in each of the units lay within the range of their specific International Pharmacopoeia or United States Pharmacopoeia monograph test for assay (percentage of the label claim) specifications;^48–52^ see Table S1.

Typically, pharmacopoeial content and uniformity methods require at least 20 units for content analysis and at least 10 units for stage one dose uniformity analysis^53^ Using a lower number of units may thus under- or over-estimate the conclusion. Since the number of units collected were limited and given the heterogeneity of within-specification threshold ranges between the different pharmacopoeias (Table S1), we also categorized each unit using the 85.0%–115.0% and the 75.0%–125.0% API threshold ranges.

Packaging analysis was conducted in comparison with the genuine medicine when these were available, blinded to chemistry and *vice versa*, by visual inspection and using the U.S. FDA CD-3.^54^ The CD-3 is a handheld device that uses different wavelengths of light to compare an authentic medical product and packaging with a potentially falsified medicine and its packaging.

As 31% of samples were analysed after their expiry date, to better understand differences in %API in relation to the medicine’s expiry date, a regression analysis of %API versus days until expiration (defined as expiry date – date of analysis) was conducted using a two-parameter decay equation [y = a×exp(–bx), y = %API, x = days till expiration]. Sigmaplot 12.0 was used to calculate the non-parametric Spearman correlation value and the associated *P* value. The %API was normalized using the rate constant ‘b’ from the equation to compensate for any possible API degradation due to analysing samples past their expiry date.

This report has been written following the Medicine Quality Assessment Reporting Guidelines (MEDQUARG), and the results have been reported to the Lao Food and Drug Department (FDD) and WHO RapidAlert.^55–57^

### Ethics

Ethical clearance was granted by the Lao PDR National Ethics Committee for Health Research (NECHR); approval reference number 054.

## Results

### Survey description

A total of 147 outlets were sampled in the 10 districts: 45 in the rural and 102 in the urban districts. Three outlets were closed and therefore 144 (98%) were included in the analysis (Figure S1 and Figure S2).

Registered outlets accounted for 97.2% (140) of those included. Pharmacy classes I and II accounted for 30.9% (43) and 30.9% (43) of outlets, respectively, and 33.8% (47) were pharmacy class III.^31,46^ Only 4.1% (6) of outlets were registered clinics, and one of the outlets sampled was a shop of a registered pharmaceutical manufacturer, Pharmaceutical Factory No. 2. Only four unregistered outlets (2.7%) were found, and they were general shops that were also selling medicines.

Antibiotics were bought from 96.5% (139) of the included outlets. Mystery shoppers were unable to buy antibiotics in five outlets. The provider was absent in one outlet and four did not have antibiotics in stock.

No provider requested to see a medical prescription.

### Medicines offered to mystery shoppers

A total of 1173 medicine samples were collected and, of those, 158 were antimalarials and 1015 were medicines for the treatment of fever and TB and not for malaria.

Out of the 1015 medicine samples sold for the treatment of fever (as claimed by outlet staff), 95.5% (969) were labelled as antibiotics and 4.5% (46) were labelled as other types of medicines such as paracetamol, antihistamines and vitamins (Figure S3 and Table S2).

Of the antibiotics collected, 15.6% (151/969) of samples were sold as loose units of only one type of medicine in plastic bags with no label or patient information, stated manufacturer and expiry date, and, of those, 84.7% (128) had no trade name and 47.7% (72) had no dosage information. Of these 128 plastic bags, 88.3% (113) capsule samples were sold as containing tetracycline, 3.1% (4) capsule samples as containing doxycycline, 2.3% (2 tablets and 1 capsule) as ampicillin, 1.6% (2) samples as ciprofloxacin tablets, 1.6% (2) as chloramphenicol capsules, 1.6% (2) as isoniazid tablets, 0.7% (1) as amoxicillin capsules and 0.7% (1) as ofloxacin tablets. Only 9.6% (93/969) of the samples were sold with secondary packaging (i.e. boxes or containers) and 11.8% (115/969) of samples gave storage instructions on the package or in a leaflet.

Eight (0.8%) samples had expired at the time of sample collection and a further 308 (31.8%) by the time chemical analysis was conducted.

Ampicillin (26.5%, 257) and amoxicillin (23.4%, 227) were the medicines most frequently sold, accounting for half of the antibiotics collected. Other antibiotics collected include sulfamethoxazole/trimethoprim (12.9%, 125), tetracycline (12.1%, 117), doxycycline (9.0%, 87), ofloxacin (7.0%, 68), ciprofloxacin (1.9%, 18), cephalexin (1.2%, 12), norfloxacin (1.2%, 12), IV ceftriaxone (1.0%, 10) and four (0.4%) samples of chloramphenicol (Table S2).

Anti-TB monotherapies were collected in 13 (9.0%) outlets, consisting of 10 (1.0%) samples of rifampicin, 6 (0.6%) samples of isoniazid and 4 (0.4%) samples of ethambutol. These were not analysed chemically.

CD-3 analysis could be conducted on 345 samples as genuine comparators were not available for 62.0% (564) of the 909 medicines selected for analysis. Of those, 56.2% (194) of the samples failed packaging analysis; 61.2% (71) of the tetracycline samples collected had the same tablet design and were consistent with each other under the CD-3 light, but were not consistent when analysed against the only genuine comparator available. No correlation between failing visual inspection and failing chemical analysis was found (*P* = 0.056).

A total of 25.11% (57) of the amoxicillin, 16.9% (7) of the sulfa-methoxazole/trimethoprim, 15.5% (40) of the ampicillin, 13.7% (12) of the doxycycline, 11.1% (2) of the ciprofloxacin and 7.3% (5) of the ofloxacin samples failed packaging analysis.

### Chemical quality of the antibiotics

Of the 969 antibiotics collected, 909 samples and nine APIs were analysed, including amoxicillin, ampicillin, ceftriaxone, ciprofloxacin, doxycycline, ofloxacin, sulfamethoxazole, tetracycline and trimethoprim; 60 samples stated as containing other APIs were not analysed (Figure S3 and Table S3).

Out of the 1034 APIs analysed (909 samples + 125 trimethoprim from the co-formulated sulfamethoxazole/ trimethoprim), 9 samples were lost during analysis. All the 1025 API samples analysed labelled as antibiotics contained the stated API.

**Figure 1:**
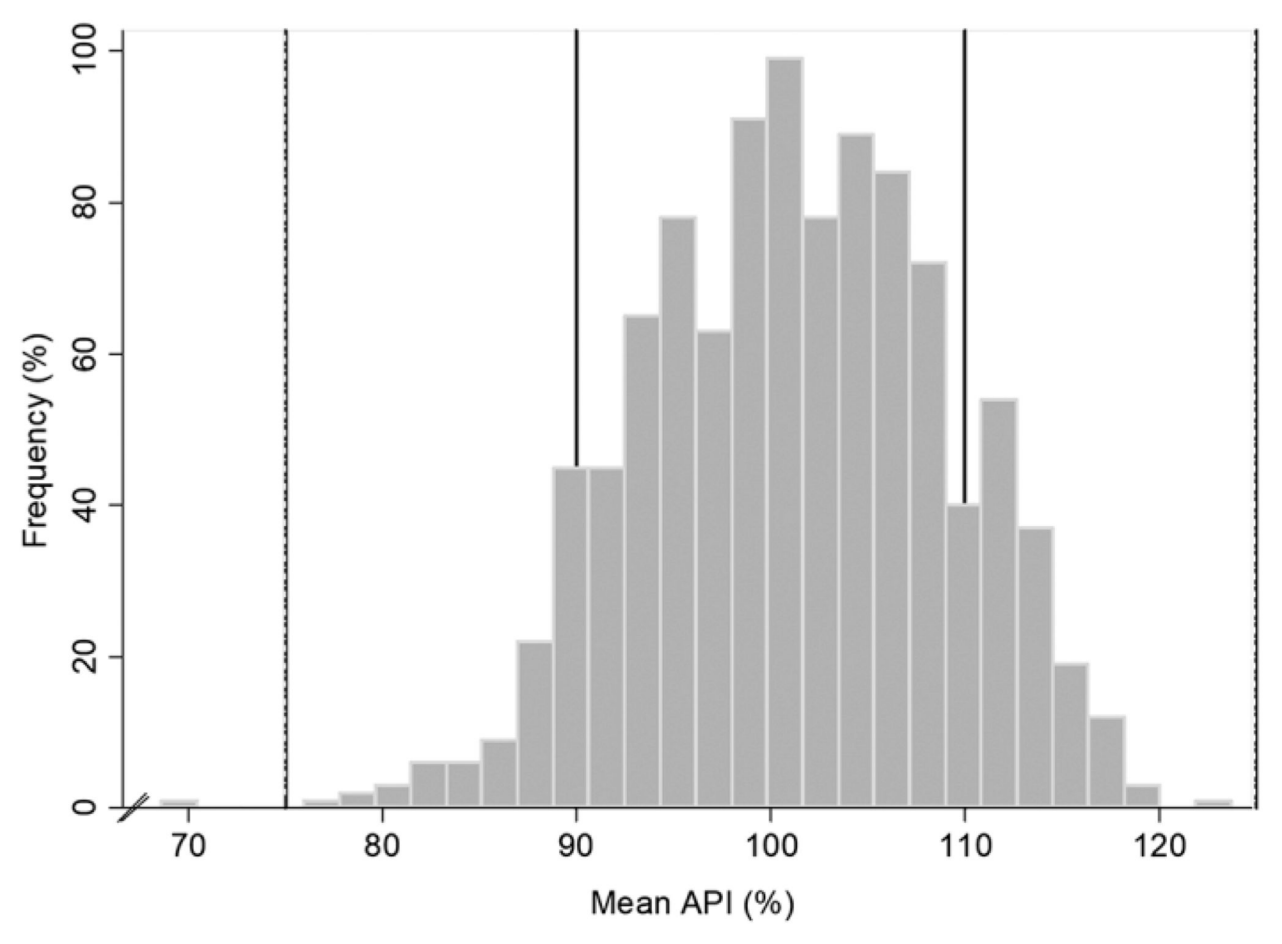
Frequency of antibiotic mean %API found in the samples (*n* = 1025). The two outer lines represent 75% and 125% cut-offs and the two inner lines represent 90% and 110% pharmacopoeial %API limits.

**Figure 2:**
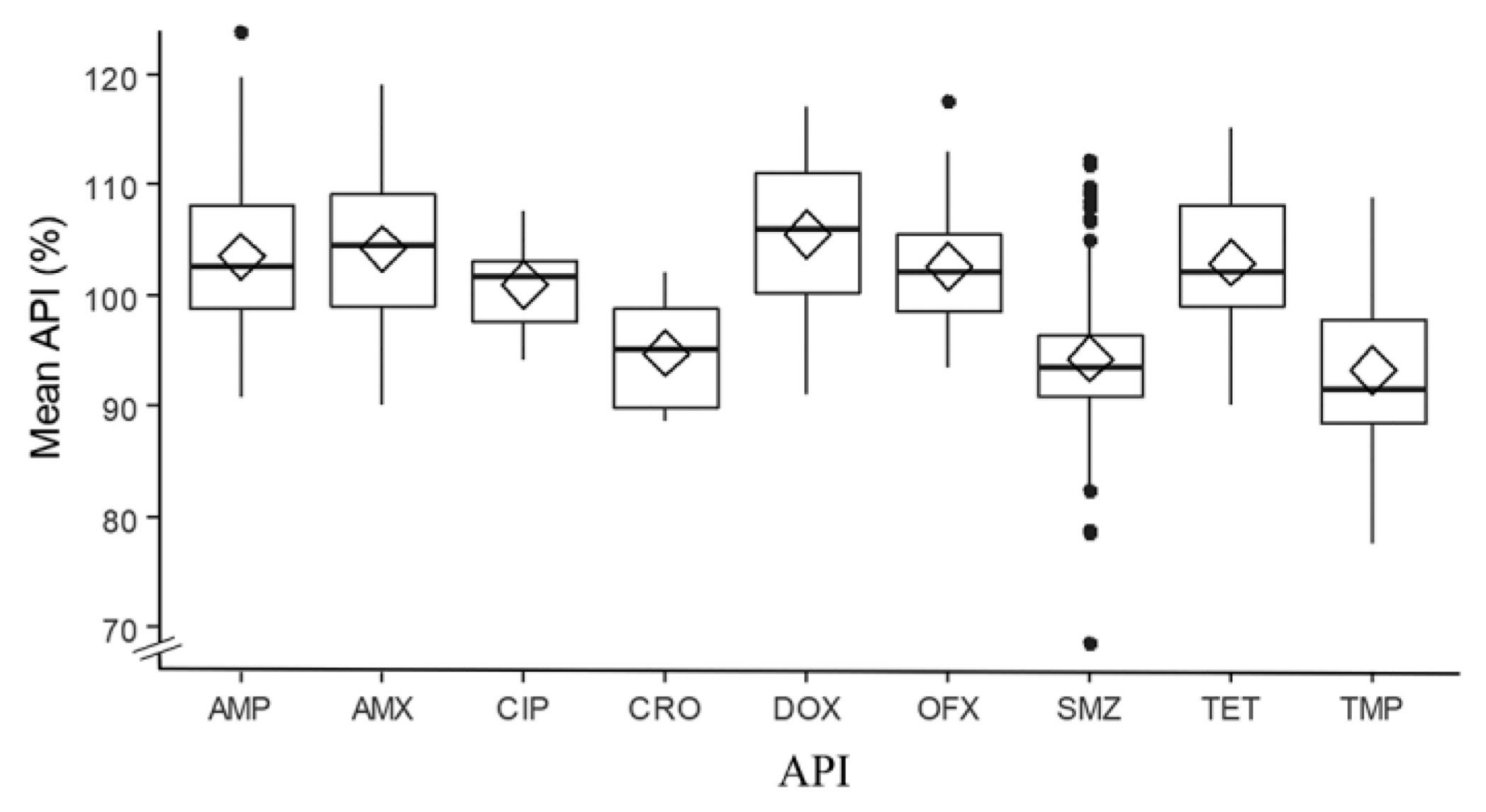
Box plot showing the mean API (%) by medicine. It includes both IV and oral forms. Diamonds represent mean values and horizontal lines represent median values. Box borders represent the lower and upper quartiles (25th and 75th percentiles, respectively). AMP, ampicillin (*n* = 256); AMX, amoxicillin (*n* = 225); CRO, ceftriaxone (*n* = 7); CIP, ciprofloxacin (*n* = 17); DOX, doxycycline (*n* = 87); OFX, ofloxacin (*n* = 68); SMZ, sulfamethoxazole (*n* = 124); TET, tetracycline (*n* = 117); TMP, trimethoprim (*n* = 124).

All except one sample had all the units tested with %API between 75% and 125% of the content stated on the label. Most of the samples (96.1%, 985) had their units’ mean %API between 85% and 115%, and 80.4% (824) had their units’ mean between 90% and 110% (Figures 1 and 2).

The sample with %API outside the range 75%–125% was a sulfamethoxazole/trimethoprim co-formulated syrup (stated as manufactured by PDC, Pharmaceutical Factory No. 3, Laos) in which one of the two sulfamethoxazole units analysed had a %API below the 75% cut-off (API values: 60% and 77% per sulfamethoxazole unit tested; 82% and 102% per trimethoprim unit tested).

In total, 81.7% (837) of the samples had all their units within the API-specific limit ranges of the International Pharmacopoeia/United States Pharmacopoeia assay specifications (% label claim) (Tables 1 and 2 and Table S1).

Amoxicillin in capsule form, ampicillin in capsule, tablet and syrup forms, ciprofloxacin hydrochloride tablets, and tetracycline in capsule and tablet forms had all their units within the International Pharmacopoeia/United States Pharmacopoeia specifications for %API-specific limit ranges (Table 1).

Both samples of sulfamethoxazole in syrup form were outside the International Pharmacopoeia specifications for %API-specific limit ranges.

Of the 124 co-formulated sulfamethoxazole/trimethoprim samples analysed, 42.7% (53) had both APIs within the limit range, 33.1% (41) of samples failed for both APIs and 24.2% (30) of samples failed for either sulfamethoxazole or trimethoprim. Of the failing samples, all but five sulfamethoxazole samples had lower amounts of API than recommended by the pharmacopoeia.

Of the 188 samples (18.3% of the total tested) that were outside the specifications for assay (% label claim), 76 (40.4%) had units with higher amounts of API and 112 (59.6%) had units with lower amounts of API (Table 2).

**Figure 3:**
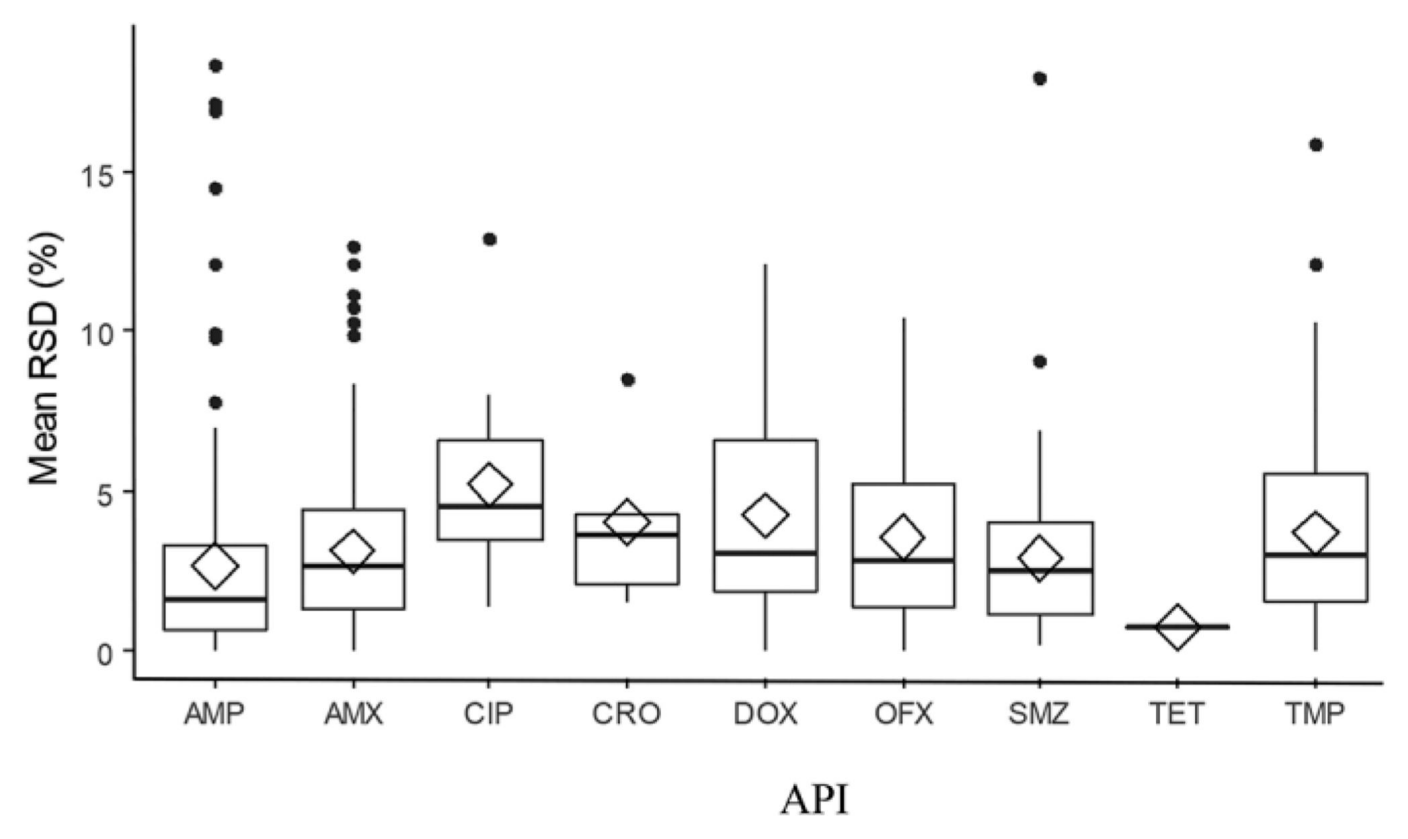
Interunit variability between units per sample measured as the RSD by medicine. It includes both IV and oral forms. Diamonds represent mean values and horizontal lines represent median values. AMP, ampicillin (*n* = 256); AMX, amoxicillin (*n* = 225); CRO, ceftriaxone (*n* = 7); CIP, ciprofloxacin (*n* = 17); DOX, doxycycline (*n* = 87); OFX, ofloxacin (*n* = 68); SMZ, sulfamethoxazole (*n* = 124); TET, tetracycline (*n* = 117); TMP, trimethoprim (*n* = 124).

Inter-tablet variability was also measured for up to three tablets per dosage units from the same sample. Ciprofloxacin had the highest variability between its units, with mean relative standard deviation (RSD) of 5.2 (Figure 3 and Table S4 and Table S5).

There were 145 branded products labelled as from 41 stated manufacturers, of which 25.5% (37) were registered with the Lao Food and Drug Department using their list of 2012. Of the 86.4% (886) of antibiotic samples that specified a manufacturer, 25.3% (259) were labelled as made by ‘Codupha-Lao Pharmaceutical Factory, Vientiane, Lao P.D.R.’, 19.2% (197) by ‘CBF Pharmaceutical Factory, Pakse-Champasack, Lao P.D.R.’ and 12.8% (132) by ‘KPN Pharma Co., Ltd, Vientiane Lao P.D.R.’

Samples were labelled as manufactured in seven countries, with most of them (58.7%, 602) labelled as being made in Laos. Other countries stated as the origin were India (11.5%, 118), China (7%, 72), Thailand (4%, 41), Vietnam (3.7%, 38), South Korea (0.2%, 2) and Bangladesh (0.1%, 1). For 14.7% (151) of the samples, the country of manufacture was not stated.

A significant difference was found between the stated country of manufacture and the %API of the samples (Kruskal–Wallis *P* = 0.0001); 75% (141) of the failed samples were labelled as being made in Laos, 8% (15) from China, 6.4% (12) from Thailand, 5.3% (10) from India and 2.6% (5) from Vietnam, and 2.6% (5) with missing manufacturer information. The %API was significantly associated with the stated manufacturer (Kruskal–Wallis *P* = 0.0001).

A third of the samples stated as manufactured in Thailand were outside the limit range (29.3%), and 23.4% and 20.8% of the samples labelled as manufactured in Laos and China, respectively.

### Stability

**Figure 4:**
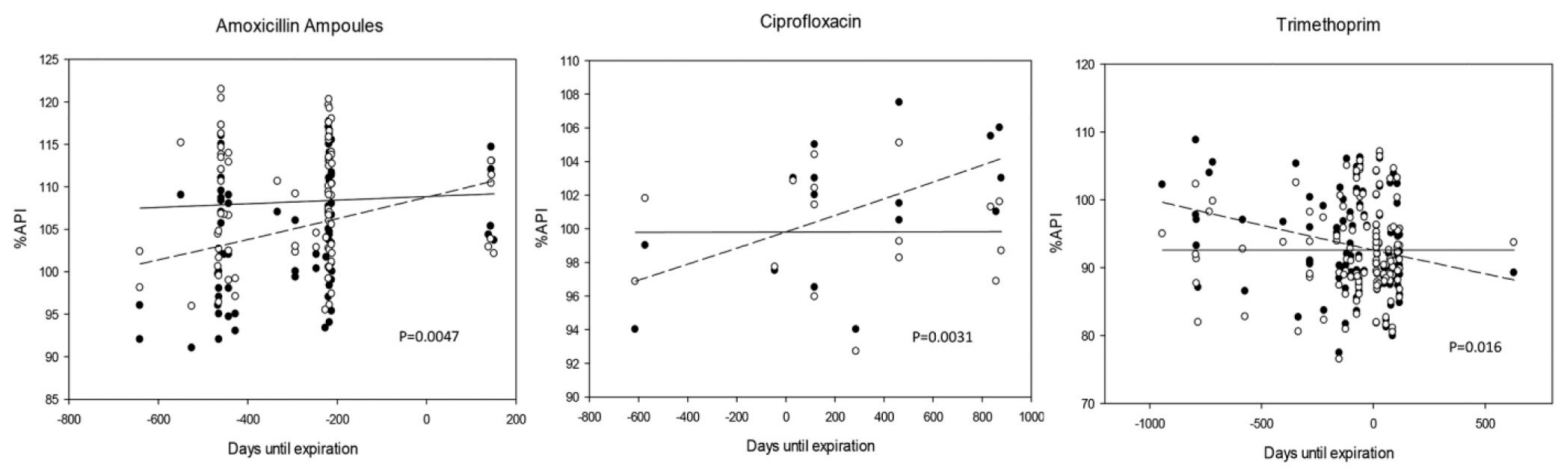
Stability plots showing changes in API content relative to expiration date. Dashed lines represent trends associated with these changes. The %API of medicines with significant slopes (*P* < 0.05) were normalized and are represented by the solid lines. Amoxicillin ampoules, *n* = 246; ciprofloxacin, *n* = 51; trimethoprim, *n* = 372.

Stability plots revealed trends in API degradation with days until expiration. Characterization of the variation of the %API in relation to the medicine’s remaining time to expiration was determined by regression analysis (Figure 4).

In this figure, dashed lines represent time trends associated with degradation in %API content. The %API of medicines with significant slopes (*P* < 0.05) were normalized by adjusting the slope to zero to compensate for these changes and is represented by the solid line.

Stability plots demonstrate changes in %API in time before and after the expiry date. Trimethoprim, ciprofloxacin and amoxicillin ampoules showed a weak to moderate correlation between %API reduction and expiration date (*P* < 0.05; Figure 4). For ciprofloxacin tablets and amoxicillin ampoules, %API significantly declined with increased sample age, but the reverse was found for trimethoprim.

## Discussion

Despite their key importance for treating infections, little is known in the public domain about the quality of antibiotics in the Greater Mekong subregion, notwithstanding the significant anecdotal evidence that poor-quality antibiotics are present in southeast Asia and elsewhere.^11,12,24,28,29,34,58,59^

All samples contained the stated API and most of the samples contained the correct mean amount of API, although there was a significant variation in the quantity of active ingredient within the samples. Results obtained from these antibiotics are consistent with the findings of the quality of antimalarials collected in the same survey.^37^

There was only one sample (sulfamethoxazole/trimethoprim co-formulated syrup) for which the dosage units contained %API <75%, but there was no genuine comparator available to ascertain the authenticity of the sample; whether it was substandard or falsified cannot therefore be confidentially ascertained.

The main problem identified was the presence of probably sub-standard, rather than falsified, antibiotics; 18.3% of the samples had units outside the International Pharmacopoeia/United States Pharmacopoeia test for assay (% label claim) specifications. In the absence of chemical assays to distinguish degradation from poor factory production in field-collected samples, it is very difficult to distinguish failed samples as degraded or substandard, or both.^32,60^ There is an urgent need for research to develop such techniques. MS fingerprinting of degradation products may allow this distinction.^61,62^ An additional issue is that smaller companies may not have the human capacity, equipment and consumables to check the quality of the imported bulk API. For substandard medicines, the quality defect may have been in the API producer rather than the factory formulating the finished product. The storage conditions until the time of purchase are unknown. Poor storage conditions may have contributed to degradation of APIs and excipients. Changes in crystalline morphology caused by high temperature can affect the dissolution or disintegration of the active ingredients, impairing bioavailability.^63–66^ This is also true when analysing expired samples, as the medicine may or may not maintain its potency beyond the expiry date. Stability plots demonstrated potency before and after the expiry date and show the variability associated with the analysis as well as the tablets.

These data are important for individual patients as they imply risk of impaired therapeutic efficacy and/or adverse drug reactions. More than a third of medicines outside the specification range had a high %API concentration. This is of particularly great concern for medicines with narrow therapeutic indices, such as chloramphenicol, which can cause bone marrow toxicity and even death. Tetracycline may be degraded under unfavourable storage conditions, resulting in potential noxious degradation products.^31,34^ Even if clinical consequences are relatively rare, they would be very difficult to detect and manage in rural Laos.^67^

A significant minority of the samples (15.6%) were sold loose with no labelling or manufacturing information. This finding is consistent with other surveys conducted in Laos.^31,35^ Inadequate labelling not only results in poor information on drug use, for patients and health workers, but it also provides opportunities for the sale of unregistered and falsified medicines.^68^ Eight samples had expired at the time of survey when there should have been none.

Antimicrobials with low %API, poor bioavailability or degradation may engender drug resistance. Modelling studies suggest that high β-lactam antibiotic doses at low frequencies produced more highly resistant *Streptococcus pneumoniae* strains, but at far lower prevalence than repeated exposure to subtherapeutic doses, which resulted in the highest prevalence of resistant strains.^7,69^

In addition, unregulated provision of antibiotics, dispensing of insufficient doses and the reduced adherence to complete dose regimens may contribute to the spread of antibiotic resistance.^70^ The high over-the-counter availability of antibiotics found in this survey suggests that overuse of antibiotics may be common. This problem becomes increasingly complex as many medicine sellers have not been trained in diagnosis and have limited knowledge on antibiotic posology and resistance.

Furthermore, anti-TB medicines, such as single agent isoniazid and rifampicin, were also sold by some outlets, even though fixed-dose combination therapy for TB is available for free for patients through the Global Fund via the National TB Programme. The unregulated use of these monotherapies is likely to precipitate treatment failure and engender TB multidrug resistance.

Accurate prescribing decisions, appropriate treatment and rational use of drugs are major concerns among healthcare services in Laos.^41,71^ Nevertheless, enhanced pharmacy regulation, health education programmes and improvements in medicine labelling in Lao language are needed to promote appropriate antibiotic use.

### Limitations

Itinerant drug sellers were not included, but they may stock anti-infective medicines and may reach remote communities. As the sale of medicines from unlicensed outlets is illegal, we may have underestimated these sources. We did not examine the quality of antibiotics in the public sector.

Dissolution and disintegration tests were not performed, and the numbers of dosage units collected per sample were low. Medicines sold in small unlabelled plastic bags (‘yaa chut’) may have already expired at the time of sample collection and the storage conditions of the samples before collection are not known. That 31% of samples were analysed after their expiry date is an important limitation and cautions against overinterpretation of these data. With the significant human investment needed to analyse samples, conducting analysis of many units before their expiry date is problematic; few papers describe date of analysis in relation to sample expiry date.

The differences found in packaging analysis suggest that samples may have been from a different manufacturer, brand, or batch. Excipient variation between different manufacturers or the coating of the samples may have had an impact; or medicines may have been degraded due to poor storage conditions. Dose to dose variations within the samples were found, and it is unclear as to how many failed dosage units in a sample should be regarded as minimally acceptable.^72^

## Supplementary Material

Suppplementary File

## Figures and Tables

**Table 1:** Quality of the samples surveyed classified by the International Pharmacopoeia/United States Pharmacopoeia assay (percentage of API label claim) limit range

Labelled as API	Number ofsamples	Dosage	Form	Number of samples lost	Ph. Int., Ninth Edition, 2019	USP 2020	Number of failed samples	% Failure
Amoxycillin anhydrous	15	1g	Ampoule		NA	90%-120%	1	6.7
Amoxycillin sodium	56	1g	Ampoule		NA	90%-120%	0	0
Amoxycillin	11	1g	Ampoule		NA	90%-120%	1	9.1
Amoxycillin	2	250mg	Capsule		NA	90%-120%	0	0.0
Amoxycillin	137	500mg	Capsule		NA	90%-120%	0	0.0
Amoxycillin	2	NA	Capsule		NA	90%-120%	0	0.0
Amoxycillin trihydrate	2	500mg	Capsule		NA	90%-120%	0	0.0
Amoxycillin	0	125mg	Syrup	1	90%-120%	90%-120%	-	-
Amoxycillin trihydrate	0	60 mL	Syrup	1	90%-120%	90%-120%	-	-
Ampicillin	5	1g	Ampoule		90%-110%	90%-115%	1	20.0
Ampicillin sodium	104	1g	Ampoule	1	90%-110%	90%-115%	20	19.2
Ampicillin	1	NA	Capsule		NA	90%-120%	0	0.0
Ampicillin	133	500mg	Capsule		NA	90%-120%	0	0.0
Ampicillin trihydrate	1	500mg	Capsule		NA	90%-120%	0	0.0
Ampicillin	10	250mg	Tablet		NA	90%-120%	0	0.0
Ampicillin	1	NA	Tablet		NA	90%-120%	0	0.0
Ampicillin	1	125mg	Syrup		NA	90%-120%	0	0.0
Ceftriaxone	3	1g	Ampoule	1	90%-110%	90%-115%	2	66.7
Ceftriaxone sodium	4	1g	Ampoule	2	90%-110%	90%-115%	1	25.0
Ciprofloxacin	15	500mg	Tablet		NA	90%-110%	2	13.3
Ciprofloxacin	1	NA	Tablet	1	NA	90%-110%	0	0.0
Ciprofloxacin hydrochloride	1	500mg	Tablet		NA	90%-110%	0	0.0
Doxycycline	80	100mg	Capsule		90%-110%	90%-120%	36	45.0
Doxycycline	5	NA	Capsule		90%-110%	90%-120%	1	20.0
Doxycycline hyclate	2	100mg	Capsule		90%-110%	90%-120%	1	50.0
Ofloxacin	68	200mg	Tablet		NA	90%-110%	10	14.7
Sulfamethoxazole^a^	122	400mg/80mg	Tablet	1	90%-110%	93%-107%	46	37.7
Sulfamethoxazole	2	200mg/40mg	Syrup		90%-110%	90%-110%	2	100.0
Tetracycline	57	250mg	Capsule		NA	90%-125%	0	0.0
Tetracycline	58	NA	Capsule		NA	90%-125%	0	0.0
Tetracycline hydrochloride	1	500mg	Capsule		NA	90%-125%	0	0.0
Tetracycline	1	NA	Tablet		NA	90%-125%	0	0.0
Trimethoprim^a^	122	400mg/80mg	Tablet	1	90%-110%	93%-107%	63	51.6
Trimethoprim	2	200mg/40mg	Syrup		90%-110%	90%-110%	1	50.0
Total	1025			9			188	18.3

Ph. Int., International Pharmacopoeia; USP, United States Pharmacopoeia; NA, not available.

a Co-formulated sulfamethoxazole and trimethoprim are counted as separate APIs.

**Table 2:** Units within, above and below the International Pharmacopoeia/United States Pharmacopoeia assay (percentage of label claim) specifications

Labelled as API	Number of samples	Failed samples		Good quality		Samples with high API		Samples with low API	
		no.	%	no.	%	no.	%	no.	%
Amoxicillin	225	2	0.9	223	99.1	0	0.0	2	0.9
Ampicillin	256	21	8.2	235	91.8	21	8.2	0	0.0
Ceftriaxone	7	3	42.9	4	57.1	0	0.0	3	42.9
Ciprofloxacin	17	2	11.8	15	88.2	2	11.8	0	0.0
Doxycycline	87	38	43.7	49	56.3	38	43.7	0	0.0
Ofloxacin	68	10	14.7	58	85.3	10	14.7	0	0.0
Sulfamethoxazole	124	48	38.7	76	61.3	5	4.0	43	34.7
Tetracycline	117	0	0.0	117	100.0	0	0.0	0	0.0
Trimethoprim	124	64	51.6	60	48.4	0	0.0	64	51.6
Total number tested	1025	188	18.3	837	81.7	76	7.4	112	10.9
Total number failed	-	188	-	-	-	76	40.4	112	59.6
