# Supplementary material for: A random survey of the prevalence of falsified and substandard antibiotics in the Lao PDR
Source: J Antimicrob Chemother. Author manuscript; Available in PMC 2023 Mar 23. (PMC7614350; doi:10.1093/jac/dkab435)
Supplement: Suppplementary File [file EMS171924-supplement-Suppplementary_File.docx]

**Supplementary data**

**Figure S1**. Map of the districts selected, and outlets sampled during the 2012 survey in southern Laos. The numbers in red refer to the number of outlets sampled within each district.

**Text S1**. Mystery shopper scenario.

**Form S1**. Anti-infective Lao Random Survey 2012 Questionnaire

**Table S1**. International Pharmacopoeia and US Pharmacopoeia specific cut offs for quality (assay limit range (percentage of label claim)).

**Figure S2**. Number of outlets included in the survey of anti-infective quality in Laos.

**Figure S3**. Number of antibiotics collected and analysed.

**Table S2**. Active Pharmaceutical Ingredients (APIs) of medicines collected during the survey.

**Table S3**. Description of the anti-infective medicines sampled in the 2012 survey.

**Table S4**. Relative standard deviation within dosage units of the anti-infective samples.

**Table S5**. Sample mean HPLC measurement (%API), SD and Mean RSD found by API (RSD is measured by API & not within the sample- all units together).

**Figure S1**. Map of the districts selected, and outlets sampled during the 2012 survey in southern Laos. The numbers in red refer to the number of outlets sampled within each district (N=147).


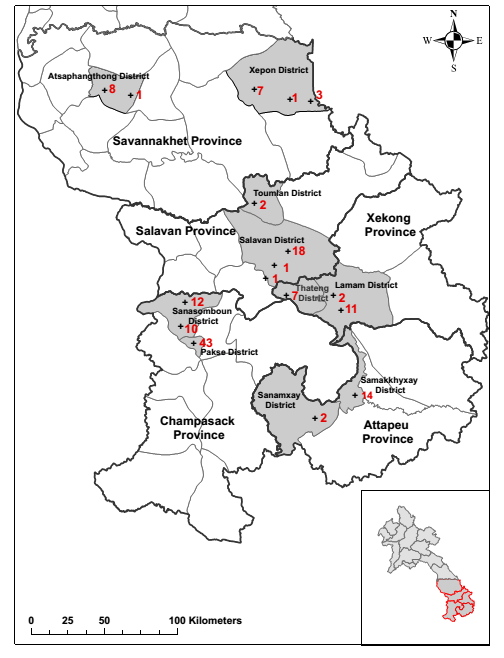


**Text S1**. Mystery shopper scenario.

The mystery shoppers visited each selected private provider dressed as a Lao manual worker stating in Lao language: *‘I would like to buy some drugs for my family – we are travelling - here is a list – may I see which ones you have so I can choose?’* The mystery shopper then enquired: *‘Do you have any other brands, please? What is this drug for? Is this drug good for infection? Do you have a stronger drug for infection?’*

**Form S1**. Anti-infective Lao Random Survey 2012 Questionnaire.

## ANTIINFECTIVE LAO RANDOM SURVEY 2012 |__|-|__|__|__|__|-|__|__|

MS District Outlet

| Section A: Provider identification information  *Instructions (See SOP for details)* | | |
| --- | --- | --- |
| A1. Today’s date (dd/mm/yyyy)  [___\|___]-[___\|___]-[ 2 _\| 0_ \| 1 _\| 2 ] | A2. Time when mystery shopper visited the outlet  [___:___] am/pm | |
| A3. Mystery Shopper’s name | A4. Mystery Shopper’s code  [___] | |
| A5. District Name | A6. District Code  [___\|___\|___\|___] | |
| A7. Name of outlet *{if no name, record “no name” or owner’s name}* | A8. Outlet code  [___\|___] | |
| A9. Research assistant code  [___\|___] | A10. Time when debriefing was done  [___:___] am/pm | |
| A11. Name of village or town | | |
| A12. Physical address or location identifiers of outlet *(detailed description that identify the outlet)* | | |
| ***A13.GPS code:*** \|**__**\|**__**\|**__**\|  Latitude \|**__**\|**__**\|**__**\|\|**__**\|**__**\|**__**\|\|**__**\|**__**\|**__**\| Longitude \|**__**\|**__**\|**__**\|\|**__**\|**__**\|**__**\|\|**__**\|**__**\|**__**\| | | |
| A14. Shop attendant   1. Male 2. Female | | \|__\| |
| A15. Interview result   1. Interview completed 2. Interview interrupted and not complete   3. Outlet not open at the time (try to make 3 attempts)  4. Provider refused to participate (why?)_________________________________ | | \|__\| |
| A16. Type of Outlet   1. Pharmacy type 1 2. Pharmacy type 2 3. Pharmacy type 3 4. Clinic 5. Drug shop 6. Other _________________________________ | | \|**__**\| |
| A17. Signposting/ Warning about Counterfeit Medicine   1. Yes, WHO “Cobra” warning sign visible 2. No 3. Other__________________________________ | | \|**__**\| |
| A18. Where are the drugs stored?   1. Cabinet inside shop 2. On open shelf (not in cabinet) 3. In the closed drawer 4. Plastic bag/basket/container 5. Other_________________________________ | | \|__\| |

| Section B. Screening Questions and Story *(circle an answer)* | | | | | |
| --- | --- | --- | --- | --- | --- |
| Stated Question  **‘I would like to buy some drugs for my family– we are travelling - here is a list – may I see which ones you have so I can choose?’** | | | | | |
| B1. **STORY:** *Ask the mystery shopper to describe any deviation from the original question* | | | | | |
| B2. Any other observations about the provider or about the interaction: | | | | | |
| Section C. Consultation *(multiple answers possible)* | | | | |  |
| C1. Approximately how long did the whole procedure take?  (estimated time) ________________minutes | | | [___] | |  |
| C2. Changes / referrals *(One answer only)*   1. All the procedure was done with the same person 2. I was attended by more than 1 person in the same outlet 3. I was referred another person in another place 4. I was referred to another clinician / health provider   Who? (name, place) ____________________ | | | [___] | |  |
| C3. What did provider advise or do initially (*one answer only)*   1. Offered me some medicine **without** asking any further questions 2. Asked me some question, examined me or offered test before offering medicine or offered/advised or asked about blood test ***(SKIP to C5)*** 3. Other (specify): _____________________________________ | | | [___] | |  |
| C4. What did you do? *(multiple response allowed)* ***(SKIP to section D)***   1. I bought the medicines he/she offered 2. I asked if he/she sold a type of medicine (specify)_______________ 3. I gave more information (eg I had malaria before) (specify)_________________   ________________________________________________________________ | | | [___] | |  |
| C5. What further questions did he/she ask you? *(multiple response allowed)*   1. No questions 2. About what kind of medicine I wanted? (what did you answer?)   _______________________________________________________   1. About what kind of medicine I already tried? (what did you answer?)   _______________________________________________________   1. About where I had been working/travelling? (what did you answer?)   _______________________________________________________   1. About whether I had malaria before? (what did you answer?)   ________________________________________________________   1. About other kinds of symptoms? (what did you answer?)   ________________________________________________________   1. Other (specify)____________________________________________ | | | [___] | |  |
| C6. Did he/she **ask** whether you already had blood test before offering medicine?   1. No 2. Yes | | | [___] | |  |
| C7. Did he/she **suggest or offer** that you have a blood test?   1. No *(Skip to Section D)* 2. Yes, suggested a test and offered to do it himself/herself 3. Yes, suggested a test but did not offer to do it | | | [___] | |  |
| Section D: Medicines | | | |  |  |
| D1. Were you able to buy any medicines? (MUST ANSWER!)   1. Yes 2. No*, why not? __________________________________ (END debriefing)* | [___] | | |  |  |
| D2. Were you asked how much money you wanted to spend or how many tablets you wanted? *(Multiple responses allowed)*   1. No 2. Yes, I was asked how much **money** I wanted to spend 3. Yes, I was asked how many **days** treatment I wanted 4. Yes, I was asked how many **packets or tablets** I wanted | [___] | | |  |  |
| D3. How many medicines did you buy in total? | [___] | | |  |  |
| D4. Were you told how to take the medicines? ie route, dose, duration?  1. Yes (specify)_______________________________________  2. No | [___] | | |  |  |
| D5. How much did you pay in total for all the medicines?    [___] [___][___][___][___][___][___] *Kip* | | | |  |  |
| D6. Did you buy any Yaa Chud?  1. Yes, Code: \|__\|-I__\|__\|__\|__\|-\|__\|__\|-\|__\|  2. No, why? _______________________________ | [___] | | |  |  |
| D7. Did the provider explain the content of Yaa Chud   1. Yes ___________________________________ 2. No | [___] | | |  |  |
| D8. How much did you pay for this medicine?    [___][___][___][___][___][___][___] *Kip* | | | |  |  |
| D9. Where you told how to take the medicine?  1. Yes ___________________________________  2. No | | [___] | |  |  |

**Table S1.** International Pharmacopoeia and US Pharmacopoeia specific cut offs for quality (assay limit range (percentage of label claim)).

| **Labelled as Active Pharmaceutical Ingredient** | **Dosage** | **Form** | **International Ph Ninth Edition, 2019** | **USP 2020** |  |
| --- | --- | --- | --- | --- | --- |
|  |  |  |  |  |  |
|  |  |  |  |  |  |
| Amoxycillin anhydrous | 1g | Ampoule | N/A | 90%–120% |  |
| Amoxycillin Sodium |  |  |  |  |  |
| Amoxycillin |  |  |  |  |  |
| Amoxycillin | 250mg | Capsule |  |  |  |
| Amoxycillin | 500mg | Capsule |  |  |  |
| Amoxycillin | N/A | Capsule |  |  |  |
| Amoxycillin trihydrate | 500mg | Capsule |  |  |  |
| Amoxycillin | 125mg | Syrup | 90-120% | 90%–120% |  |
| Amoxycillin trihydrate | 60mls |  |  |  |  |
| Ampicillin | 1g | Ampoule | 90-110% | 90-115% |  |
| Ampicillin Sodium |  |  |  |  |  |
| Ampicillin | N/A | Capsule | N/A | 90-120% |  |
|  | 500mg |  |  |  |  |
| Ampicillin Trihydrate |  |  |  |  |  |
| Ampicillin | 250mg | Tablet |  | 90-120% |  |
|  | N/A |  |  |  |  |
| Ampicillin | 125mg | Syrup | N/A | 90%–120% |  |
| Ceftriaxone | 1g | Ampoule | 90-110% | 90-115% |  |
| Ceftriaxone sodium |  |  |  |  |  |
| Ciprofloxacin | 500mg | Tablet | N/A | 90-110% |  |
|  | N/A |  |  |  |  |
| Ciprofloxacin Hydrochloride | 500mg |  |  |  |  |
| Doxycycline | 100mg | Capsule | 90-110% | 90-120% |  |
| Doxycycline | N/A |  |  |  |  |
| Doxycycline hyclate | 100mg |  |  |  |  |
| Ofloxacin | 200mg | Tablet | N/A | 90-110% |  |
| Sulfamethoxazole | 400mg/80mg | Tablet | 90-110% | 93-107% |  |
|  | 200mg/40mg | Syrup | 90-110% | 90-110% |  |
| Tetracycline | 250mg | Capsule | N/A | 90-125% |  |
|  | N/A | Capsule |  |  |  |
| Tetracycline Hydrochloride | 500mg | Capsule |  |  |  |
| Tetracycline | N/A | Tablet |  |  |  |
| Trimethoprim | 400mg/80mg | Tablet | 90-110% | 93-107% |  |
|  | 200mg/40mg | Syrup | 90-110% | 90-110% |  |

N/A, not available.

**Figure S2.** Number of outlets included in the survey of anti-infective quality in Laos

**Figure S3.** Number of antibiotics collected and analysed.

| Medicines sampled  **N=1015** |  |
| --- | --- |
|  |  |
|  | 46 medicines – not antibiotics |
| 969 Antibiotics |  |
|  |  |
|  | 60 medicines not tested for  API content |
| 909 samples* selected for analysis  (9 APIs) |  |
|  | 9 samples lost |
|  |  |
| APIs analyzed  **N=1025** |  |

* 909 samples including 125 co-formulated sulfamethoxazole -trimethoprim; making a total of 1,034 APIs samples.

**Table S2.** Active Pharmaceutical Ingredients (APIs) of medicines collected during the survey.

| **Antibiotics** | | | **Other type of medicines** | | |
| --- | --- | --- | --- | --- | --- |
| **API** | **No. Samples** | **%** | **API** | **No. Samples** | **%** |
| Ampicillin | 257 | 26.5 | Beberine | 1 | 2.2 |
| Amoxicillin | 227 | 23.4 | Cinnarizine | 1 | 2.2 |
| Sulfamethoxazole - Trimethoprim | 125 | 12.9 | Electrolyte Beverage | 1 | 2.2 |
| Tetracycline | 117 | 12.1 | Flunarizine | 1 | 2.2 |
| Doxycycline | 87 | 9.0 | Glucose Anhydrous | 8 | 17.4 |
| Ofloxacin | 68 | 7.0 | Hyoscine-N-butylbromide | 1 | 2.2 |
| Ciprofloxacin | 18 | 1.9 | Loperamide | 1 | 2.2 |
| Ceftriaxone | 10 | 1.0 | Mefenamic acid | 3 | 6.5 |
| Cephalexin | 12 | 1.2 | Paracetamol | 12 | 26.1 |
| Norfloxacin | 12 | 1.2 | Piracetam | 3 | 6.5 |
| Rifampicin | 10 | 1.0 | Salbutamol | 2 | 4.3 |
| Isoniazid | 6 | 0.6 | Salbutamol sulphate | 2 | 4.3 |
| Chloramphenicol | 4 | 0.4 | Simeticone | 1 | 2.2 |
| Ethambutol | 4 | 0.4 | Vitamin (Multivitamin) | 3 | 6.5 |
| Metronidazole | 3 | 0.3 | Vitamin B1,B2,B5,B6, PP | 1 | 2.2 |
| Phenoxymethyl Penicillin | 1 | 0.1 | Vitamine B | 1 | 2.2 |
| Azithromycin | 1 | 0.1 | Vitamine B1 | 2 | 4.3 |
| Cefixime | 1 | 0.1 | Vitamine C | 2 | 4.3 |
| Cefotaxime | 1 | 0.1 | **TOTAL** | **46** | **100.0** |
| Cefotaxime sodium | 1 | 0.1 |  |  |  |
| Erythromycin | 1 | 0.1 |  |  |  |
| Gentamicin sulphate | 1 | 0.1 |  |  |  |
| Gentamicin | 1 | 0.1 |  |  |  |
| Lincomycin | 1 | 0.1 |  |  |  |
| **TOTAL** | **969** | **100.0** |  |  |  |

**Table S3.** Description of the anti-infective medicines sampled in the 2012 survey.

| **Labelled as Active Pharmaceutical Ingredient** | **No. of samples** | **% per API** | **Dosage** | **Form** | **No. Samples per Brand** | **Stated Brand** | **Stated Manufacturer** | **Mean Price in USD** |
| --- | --- | --- | --- | --- | --- | --- | --- | --- |
|  |  |  |  |  |  |  |  | **Per unit (tab, vial, bottle, bag)** |
|  |  |  |  |  |  |  |  | **8341 Lao Kip≈ 1 USD** |
| Amoxycillin anhydrous | 15 | 6.6 | 1g | Ampoule | 10 | Amoxcil | CPDP BIDIPHAR | 1.39 |
|  |  |  |  |  | 1 | Amoxcil | Swiss Parenterals PVT.LTD Ahmedabad, India | 1.39 |
|  |  |  |  |  | 4 | Moxcil T.P | T.P. Drug Laboratory 1969 Bangkok, Thailand | 1.39 |
| Amoxycillin Sodium | 56 | 24.5 | 1g | Ampoule | 1 | Amoxcil | CBF PHARMACEUTICAL FACTORY. Pakse-Champasack- Lao P.D.R. | 1.39 |
|  |  |  |  |  | 35 | Amoxcil | Swiss Parenterals PVT.LTD Ahmedabad, India | 1.39 |
|  |  |  |  |  | 14 | Amoxcil | Swiss Prenterals PVT.LTD Ahmedabad, India | 1.39 |
|  |  |  |  |  | 6 | Amoxycillin sodium | Reyoung Pharmaceutical Co.,Ltd. Shandong, PRC | 1.39 |
| Amoxycillin | 11 | 4.8 | 1g | Ampoule | 1 | Amoxcil | Binhdinh pharmaceutical Medical Equipment Company Vietnam | 1.39 |
|  |  |  |  |  | 4 | Amoxcil | Swiss Parenterals PVT.LTD Ahmedabad, India | 1.39 |
|  |  |  |  |  | 6 | Amoxcil | Swiss Prenterals PVT.LTD Ahmedabad, India | 1.39 |
|  | 141 | 61.6 | 500mg | Capsule | 72 | Amoxycillin | CODUPHA - LAO PHARMACEUTICAL FACTORY, Vientiane, Lao P.D.R. | 1.39 |
|  |  |  |  |  | 20 | Amoxycillin | KPN Pharma co.,Ltd, Vientiane Lao P.D.R. | 1.39 |
|  |  |  |  |  | 1 | Amoxylin 500 | Factory No 2, Vientiane, Lao P.D.R. | 1.39 |
|  |  |  |  |  | 6 | GPO MOX | GPO, Thailand | 1.39 |
|  |  |  |  |  | 1 | Lamoxy | Lyka Labs Limited, Maharashtra, India | 1.39 |
|  |  |  |  |  | 1 | Starmox500 | Seven Stars Pharmaceutical, Nakornpatom, Thailand | 1.39 |
|  |  |  |  |  | 1 | U-Amox 500 | The United Drug (1996) Co.;Ltd, Bangkok, Thailand | 1.39 |
|  |  |  |  |  | 6 | Amoxicillin | Zhangfeng pharmaceutical factory, Longchuan, Yunnan, China | 1.39 |
|  |  |  |  |  | 1 | Amoxicillin | Kunming Baker Norton Pharmaceutical Co. Ltd., Kunming, China | 1.39 |
|  |  |  |  |  | 1 | Amoxicillin | Domesco, 66 National Road - Cao Lanh City - Vietnam | 1.39 |
|  |  |  |  |  | 27 | Amoxcin 500 | CBF PHARMACEUTICAL FACTORY. Pakse-Champasack- Lao P.D.R. | 1.39 |
|  |  |  | NOT STATED |  | 1 | M&H | M & H Manufacturing Co., Ltd., Bangkok, Thailand | 1.39 |
|  |  |  | NOT STATED |  | 1 | NOT STATED | NOT STATED | 1.39 |
|  |  |  | 250mg |  | 2 | Amoxcin 250 | CBF PHARMACEUTICAL FACTORY, Pakse-Champasack- Lao P.D.R. | 1.39 |
|  | 1 | 0.4 | 125mg | Syrup | 1 | Amoxin | CBF PHARMACEUTICAL FACTORY. Pakse-Champasack- Lao P.D.R. | 1.39 |
| Amoxycillin trihydrate | 2 | 0.9 | 500mg | Capsule | 2 | Amoxicillin 500 | PDC, Pharmaceutical Factory N.3 km9, Lao P.D.R | 1.40 |
|  | 1 | 0.4 | 60mls | Syrup | 1 | Amixin | LACHMANN Co;Ltd | 1.40 |
|  | | | | | | | | |
| Ampicillin | 151 | 58.3 | 1g | Ampoule | 1 | Ampicillin | Binhdinh pharmaceutical Medical Equipment Company Vietnam | 0.42 |
|  |  |  |  |  | 1 | Ampicillin | Laoxuang Co.,Ltd | 0.36 |
|  |  |  |  |  | 2 | Ampicillin | Swiss Prenterals PVT.LTD Ahmedabad,India | 0.48 |
|  |  |  |  |  | 1 | Lao characters (Kanya Leo Suang) | Zishuang Zhangdong, China | 0.36 |
|  |  |  | NOT STATED | Capsule | 1 | NOT STATED | NOT STATED | 0.24 |
|  |  |  | 500mg |  | 30 | Ampicin 500 | CBF PHARMACEUTICAL FACTORY. Pakse-Champasack- Lao P.D.R. | 0.06 |
|  |  |  |  |  | 64 | Ampicillin | CODUPHA - LAO PHARMACEUTICAL FACTORY, Lao P.D. R. | 0.06 |
|  |  |  |  |  | 1 | Ampicillin | Domesco, 66 National Road - Cao Lanh City Vietnam | 0.12 |
|  |  |  |  |  | 32 | Ampicillin | KPN Pharma co.,Ltd, Vientiane Laos PDR | 0.07 |
|  |  |  |  |  | 1 | Ampicillin | PDC, Pharmaceutical Factory N.3 km9, Laos | 0.06 |
|  |  |  |  |  | 1 | Ampicillin | Seven Stars Pharmaceutical, Nakornpatom, Thailand | 0.07 |
|  |  |  |  |  | 4 | Ampicillin | Zhangfeng pharmaceutical factory, Longchuan, Yunnan, China | 0.06 |
|  |  |  | 125mg | Syrup | 1 | Ampicin Dry | CBF PHARMACEUTICAL FACTORY. Pakse-Champasack- Lao P.D.R. | 0.96 |
|  |  |  | 250mg | Tablet | 6 | AMP 250 | CDP | 0.03 |
|  |  |  |  |  | 3 | AMPI 250 | KPN Pharma co.,Ltd,Vientiane Laos | 0.04 |
|  |  |  |  |  | 1 | NOT STATED | NOT STATED | 0.09 |
|  |  |  | NOT STATED |  | 1 | NOT STATED | NOT STATED | 0.09 |
| Ampicillin Sodium | 105 | 40.5 | 1g | Ampoule | 10 | Ampicillin | Binhdinh pharmaceutical Medical Equipment Company Vietnam | 0.53 |
|  |  |  |  |  | 1 | Ampicillin | CBF PHARMACEUTICAL FACTORY. Pakse-Champasack- Lao P.D.R. | 0.24 |
|  |  |  |  |  | 46 | Ampicillin sodium | Guilin Pharmaceutical (Shanghai) Co., Ltd., China | 0.50 |
|  |  |  |  |  | 3 | Ampicillin sodium | Reyoung Pharmaceutical Co.,Ltd. Shandong, PRC | 0.40 |
|  |  |  |  |  | 4 | Ampicillin sodium | Shijiazhuang Pharmaceutical Group Co., Ltd. (CSPC), China | 0.39 |
|  |  |  |  |  | 1 | Ampicillin | Swiss Parenterals PVT.LTD Ahmedabad,India | 0.36 |
|  |  |  |  |  | 38 | Ampicillin | Swiss Prenterals PVT.LTD Ahmedabad,India | 0.50 |
|  |  |  |  |  | 2 | Ampicillin sodium | T.P. Drug Laboratory 1969 Bangkok, Thailand | 0.36 |
| Ampicillin Trihydrate | 1 | 0.4 | 500mg | Capsule | 1 | Ampicillin | CODUPHA - LAO PHARMACEUTICAL FACTORY, Lao P.D. R. | 0.06 |
|  | | | | | | | | |
| Ceftriaxone | 4 | 40 | 1g | Ampoule | 1 | Ceftriaxon | CBF PHARMACEUTICAL FACTORY. Pakse-Champasack- Lao P.D.R | 2.16 |
|  |  |  |  |  | 2 | Ceftrione | Swiss Parenterals PVT.LTD Ahmedabad,India | 1.32 |
|  |  |  |  |  | 1 | Ceftrione | Swiss Prenterals PVT.LTD Ahmedabad,India | 1.20 |
| Ceftriaxone sodium | 6 | 60 |  |  | 6 | Ceftriaxone | T.P. Drug Laboratory 1969 Bangkok, Thailand | 1.80 |
|  | | | | | | | | |
| Ciprofloxacin | 17 | 94.4 | 500mg | Tablet | 1 | Eurocapro | Globe Pharmaceuticals LTD Bangladesh | 0.12 |
|  |  |  |  |  | 4 | Ecoflox-500 | Medley pharmaceutical Co; Ltd., India | 0.12 |
|  |  |  |  |  | 1 | Microluss-500 | Microlabs Limited Unit III, Bangalor India | 0.06 |
|  |  |  |  |  | 1 | Ciprofloxacin Tablets USP 500mg | Minimed Laboratories Pvt. Ltd., Mumbai, India | 0.12 |
|  |  |  |  |  | 1 | Sepratis | SPM Corporation, Vietnam | 0.18 |
|  |  |  |  |  | 7 | Ciproxyl*500 | NOT STATED | 0.12 |
|  |  |  | NOT STATED |  | 2 | NOT STATED | NOT STATED | 0.07 |
| Ciprofloxacin Hydrochloride | 1 | 5.6 | 500mg |  | 1 | Cobay-500 | Millimed, Bangkok, Thailand | 0.11 |
|  | | | | | | | | |
| Doxycycline | 80 | 81.6 | 100mg | Capsule | 14 | Doxylcap | Bangkok Lab & Cosmetic Co.,Ltd., Thailand | 0.05 |
|  |  |  |  |  | 2 | Doxycycline Capsule BP | BRAWN Laboratories Fanalabad Haryana | 0.04 |
|  |  |  |  |  | 41 | Doxycycline | CODUPHA - LAO PHARMACEUTICAL FACTORY, Vientiane Lao P.D.R. | 0.06 |
|  |  |  |  |  | 3 | Doxycycline 100 | CODUPHA - LAO PHARMACEUTICAL FACTORY, Vientiane, Lao P.D.R. | 0.06 |
|  |  |  |  |  | 4 | Doxycycline | CTCP DUOC Dopharma J.S.C. Vietnam | 0.05 |
|  |  |  |  |  | 2 | Doxyl | Factory No 2, Sokpaluang Rd. P.O.Box 2580, Vientiane, LaoPDR | 0.06 |
|  |  |  |  |  | 1 | Doxycycline Capsule BP | Intas Pharmaceuticals LDT, Gujarat, India | 0.06 |
|  |  |  |  |  | 3 | Doxycycline | Mekophar MKP Chemical Pharmaceutical, Vietnam | 0.08 |
|  |  |  |  |  | 1 | Doxycycline capsule BP 100mg | Minimed Laboratories Pvt. Ltd., Mumbai, India | 0.06 |
|  |  |  |  |  | 1 | Doxycycline | Seven Stars Pharmaceutical, Nakornpatom, Thailand | 0.06 |
|  |  |  |  |  | 7 | Doxycycline | Vidipha Central Pharmaceutical Joint Stock Company, Vietnam | 0.07 |
|  |  |  |  |  | 1 | Doxycycline capsule | NOT STATED | 0.04 |
| Doxycycline | 4 | 4.1 | NOT STATED |  | 4 | NOT STATED | NOT STATED | 0.05 |
|  | 1 | 1.0 | NOT STATED |  | 1 | Doxylcap | Bangkok Lab & Cosmetic Co.,Ltd. , Thailand | 0.04 |
| Doxycycline hyclate | 2 | 2.0 | 100mg |  | 2 | Amermycin | Unison Laboratories Co.Ltd, Chachoengsao, Thailand | 0.08 |
|  | | | | | | | | |
| Ofloxacin | 68 | 100 | 200mg | Tablet | 1 | Medliflox200 | Medley pharmaceutical Co; Ltd., Kachigam, India | 0.12 |
|  |  |  |  |  | 40 | Ofloxin 200 | CBF PHARMACEUTICAL FACTORY. Pakse-Champasack- Lao P.D.R. | 0.08 |
|  |  |  |  |  | 5 | Ofloxacin | CODUPHA - LAO PHARMACEUTICAL FACTORY, Vientiane, Lao P.D.R. | 0.10 |
|  |  |  |  |  | 4 | Ofloxacin | Flamingo Pharmaceuticals, Mumbai, India | 0.09 |
|  |  |  |  |  | 2 | Ofloxacin | Mekophar MKP Chemical Pharmaceutical, Vietnam | 0.06 |
|  |  |  |  |  | 1 | Ofloxin | CODUPHA - LAO PHARMACEUTICAL FACTORY, Vientiane, Lao P.D.R. | 0.12 |
|  |  |  |  |  | 1 | Ofloxin | Factory No 2, Vientiane, Lao P.D.R. | 0.18 |
|  |  |  |  |  | 1 | Ofloxin | Mekophar MKP Chemical Pharmaceutical, Vietnam | 0.05 |
|  |  |  |  |  | 1 | Ofloxin | Zhangfeng pharmaceutical factory, Yunnan, China | 0.08 |
|  |  |  |  |  | 2 | Ofus | SCD Samchundang Pharma Korea, Seoul, South Korea | 0.30 |
|  |  |  |  |  | 1 | Seracin200 | Pharmaland | 0.10 |
|  |  |  |  |  | 1 | 200 | NOT STATED | 0.06 |
|  |  |  |  |  | 8 | Oflocee*200 | NOT STATED | 0.10 |
|  | | | | | | | | |
| Sulfamethoxazole - Trimethoprim | 125 | 100 | 400mg/80mg | Tablet | 24 | Bidiseptol | CBF PHARMACEUTICAL FACTORY. Pakse-Champasack- Lao P.D.R. | 0.04 |
|  |  |  |  |  | 22 | Biseptrim | CBF PHARMACEUTICAL FACTORY. Pakse-Champasack- Lao P.D.R. | 0.05 |
|  |  |  |  |  | 37 | Vactrim | CODUPHA - LAO PHARMACEUTICAL FACTORY, Vientiane, Lao P.D.R. | 0.05 |
|  |  |  |  |  | 2 | Sulfatrim | Factory No 2, Vientiane, Lao P.D.R. | 0.05 |
|  |  |  |  |  | 38 | Strim-Side | KPN Pharma co.,Ltd, Vientiane, Lao P.D.R. | 0.05 |
|  |  |  | 200mg/40mg | Syrup | 1 | Bactrin | PDC, Pharmaceutical Factory, Lao P.D.R. | 0.96 |
|  |  |  |  |  | 1 | Mycosamthong | S.M Pharmaceutical Co., Ltd, 10 Sukumvit, Samutprakarn, Thailand | 1.62 |
|  | | | | | | | | |
| Tetracycline | 116 | 97.5 | 250mg | Capsule | 1 | TETRA 250 CBF | CBF PHARMACEUTICAL FACTORY. Pakse-Champasack- Lao P.D.R. | 0.02 |
|  |  |  |  |  | 1 | TETRA 250 | CBF PHARMACEUTICAL FACTORY. Pakse-Champasack- Lao P.D.R. | 0.02 |
|  |  |  |  |  | 51 | TETRA 250 | NOT STATED | 0.03 |
|  |  |  |  |  | 2 | NOT STATED | NOT STATED | 0.03 |
|  |  |  | NOT STATED |  | 1 | 2 | Factory No 2, Vientiane, Lao P.D.R. | 0.06 |
|  |  |  |  |  | 57 | NOT STATED | NOT STATED | 0.03 |
|  |  |  |  |  | 2 | TETRA 250 | NOT STATED | 0.04 |
|  |  |  | NOT STATED | Tablet | 1 | NOT STATED | NOT STATED | 0.02 |
| Tetracycline Hydrochloride | 1 | 0.8 | 500mg | Capsule | 1 | GALNO | KPN Pharma co.,Ltd, Vientiane Lao P.D.R. | 0.09 |
| **Antibiotic samples not analyzed** | | | | | | | | |
| Azithromycin | 1 | 100 | 1000mg | Tablet | 1 | AZEE-1000 | CIPLA LTD INDIA | 4.20 |
| Cefixime | 1 | 100 | 400mg | Tablet | 1 | Cefix-400 | CIPLA LTD INDIA | 4.20 |
| Cefotaxime | 1 | 100 | 1g | Ampoule | 1 | Rotafaz | Rotaline Molekule PVT.Ltd, Maharashtra, India | 0.84 |
| Cefotaxime sodium | 1 | 100 | 1g | Ampoule | 1 | Fotax injection | M & H Manufacturing Co., Ltd., Bangkok, Thailand | 1.56 |
| Cephalexin | 12 | 100 | 500mg | Capsule | 5 | Cephalexin 500 | CBF PHARMACEUTICAL FACTORY. Pakse-Champasack- Lao P.D.R. | 0.10 |
|  |  |  |  |  | 1 | CEPHE-500 | CODUPHA - LAO PHARMACEUTICAL FACTORY, Vientiane, Lao P.D. R. | 0.12 |
|  |  |  |  |  | 1 | Cefalexin | CTC PDP, TV Pharm | 0.06 |
|  |  |  |  |  | 1 | Cefalexin | Domesco, 66 National Road - Cao Lanh City, Vietnam | 0.18 |
|  |  |  |  |  | 3 | Cefalexin | Zhangfeng pharmaceutical factory, Longchuan, Yunnan, China | 0.11 |
|  |  |  | 400mg | Capsule | 1 | Cefalexin | Zhangfeng pharmaceutical factory, Longchuan, Yunnan, China | 0.12 |
| Chloramphenicol | 4 | 100 | 250mg | Capsule | 1 | Cloramphenicol | VPC Pharimexco, Vinh Long city, Vietnam | 0.06 |
|  |  |  |  | Tablet | 1 | Chloramphenicol | Central pharmaceutical factory no 25 (UPHACE), Ho Chi Minh City , Viet Nam | 0.13 |
|  |  |  | NOT STATED | Capsule | 2 | NOT STATED | NOT STATED | 0.04 |
| Erythromycin | 1 | 100 | 250mg | Tablet | 1 | DI-ERY 250 | PDC, Pharmaceutical Factory N.3 km9, Laos | 0.04 |
| Ethambutol | 4 | 100 | 400mg | Tablet | 4 | Axotham 400 | Axon Drugs PVT.LTD, Chennai, Bangalore, India | 0.09 |
| Gentamicin sulphate | 1 | 100 | 2ml | Ampoule | 1 | Gentamicin sulphate | Zhangfeng pharmaceutical factory, Longchuan, Yunnan, China | 0.36 |
| Gentamicin | 1 | 100 | 80mg | Ampoule | 1 | Antamivin injection | AMNLIFE Sciences PVT LTD, Ahmedabad, INDIA | 0.60 |
| Isoniazid | 1 | 16.7 | NOT STATED | Tablet | 1 | NOT STATED | PATAR, Patar Lab Ltd., Bangkok, Thailand | 0.12 |
|  | 1 | 16.7 | NOT STATED | Tablet | 1 | NOT STATED | NOT STATED | 0.05 |
|  | 1 | 16.7 | 150mg | Tablet | 1 | INH | NOT STATED | 0.06 |
|  | 3 | 50.0 | 150mg | Tablet | 3 | MEKO INH150 | Mekophar MKP Chemical Pharmaceutical Vietnam | 0.03 |
| Lincomycin | 1 | 100 | 500mg | Capsule | 1 | Lincomycin | Domesco, Cao Lanh City, Vietnam | 0.05 |
| Metronidazole | 2 | 66.7 | 250mg | Tablet | 2 | Metronidazol | CBF PHARMACEUTICAL FACTORY. Pakse-Champasack- Lao P.D.R. | 0.09 |
|  | 1 | 33.3 | 500mg | Tablet | 1 | Metrozol 500 | CBF PHARMACEUTICAL FACTORY. Pakse-Champasack- Lao P.D.R. | 0.12 |
| Norfloxacin | 12 | 100 | 400mg | Tablet | 5 | Norflox 400 | CBF PHARMACEUTICAL FACTORY, Pakse-Champasack- Lao P.D.R. | 0.09 |
|  |  |  |  |  | 1 | Norflox 400 | NOT STATED | 0.06 |
|  |  |  |  |  | 5 | Norfloxyl*400 | NOT STATED | 0.11 |
|  |  |  |  |  | 1 | Rexacin 400 | NOT STATED | 0.12 |
| Phenoxymethyl Penicillin | 1 | 50.0 | 400000UI | Tablet | 1 | Penicilline V | CBF PHARMACEUTICAL FACTORY. Pakse-Champasack- Lao P.D.R. | 0.06 |
| Rifampicin | 3 | 30.0 | 300mg | Capsules | 3 | RIFAMINI | Minimed Laboratories Pvt. Ltd., Maharashtra, India | 0.12 |
|  | 6 | 60.0 |  |  | 6 | Rifampicin | Mekophar MKP Chemical Pharmaceutical Vietnam | 0.11 |
|  | 1 | 10.0 |  |  | 1 | Ultifort | Flamingo Pharmaceuticals, Mumbai,India | 0.18 |

**Table S4**. Sample relative standard deviation within dosage units of the anti-infectives samples.

| Active Pharmaceutical Ingredient | Mean RSD | SD RSD | No. Of Samples |
| --- | --- | --- | --- |
| Amoxicillin | 3.1 | 2.5 | 225 |
| Ampicillin | 2.6 | 3.3 | 256 |
| Ceftriaxone | 4.0 | 2.7 | 7 |
| Ciprofloxacin | 5.2 | 2.7 | 17 |
| Doxycycline | 4.2 | 3.2 | 87 |
| Ofloxacin | 3.5 | 2.7 | 68 |
| Sulfamethoxazole | 2.9 | 2.4 | 124 |
| Tetracycline | 0.7 | n/a | 117 |
| Trimethoprim | 3.7 | 2.8 | 124 |

**Table S5**. Sample mean HPLC measurement (%API), SD and Mean RSD found by API (RSD is measured by API & not within the sample).

| Active Pharmaceutical Ingredient | Mean HPLC | SD HPLC | mean RSD | No. Of Samples |
| --- | --- | --- | --- | --- |
| Amoxicillin | 104.2 | 7.4 | 7.1 | 486 |
| Ampicillin | 102.7 | 6.7 | 6.5 | 389 |
| Ceftriaxone | 93.4 | 5.8 | 6.2 | 17 |
| Ciprofloxacin | 101.0 | 5.8 | 5.8 | 33 |
| Doxycycline | 105.5 | 7.6 | 7.2 | 166 |
| Ofloxacin | 102.5 | 5.9 | 5.7 | 132 |
| Sulfamethoxazole | 94.2 | 7.1 | 7.5 | 240 |
| Tetracycline | 102.8 | 6.0 | 5.8 | 118 |
| Trimethoprim | 93.1 | 7.5 | 8.1 | 240 |
